# Where the Action Is—Leukocyte Recruitment in Atherosclerosis

**DOI:** 10.3389/fcvm.2021.813984

**Published:** 2022-01-11

**Authors:** Carina Mauersberger, Julia Hinterdobler, Heribert Schunkert, Thorsten Kessler, Hendrik B. Sager

**Affiliations:** ^1^Department of Cardiology, German Heart Center Munich, Technical University Munich, Munich, Germany; ^2^DZHK (German Centre for Cardiovascular Research), Partner Site Munich Heart Alliance, Munich, Germany

**Keywords:** atherosclerosis, vascular inflammation, leukocyte recruitment, adhesion molecules, integrin, transendothelial migration

## Abstract

Atherosclerosis is the leading cause of death worldwide and leukocyte recruitment is a key element of this phenomenon, thus allowing immune cells to enter the arterial wall. There, in concert with accumulating lipids, the invading leukocytes trigger a plethora of inflammatory responses which promote the influx of additional leukocytes and lead to the continued growth of atherosclerotic plaques. The recruitment process follows a precise scheme of tethering, rolling, firm arrest, crawling and transmigration and involves multiple cellular and subcellular players. This review aims to provide a comprehensive up-to-date insight into the process of leukocyte recruitment relevant to atherosclerosis, each from the perspective of endothelial cells, monocytes and macrophages, neutrophils, T lymphocytes and platelets. In addition, therapeutic options targeting leukocyte recruitment into atherosclerotic lesions—or potentially arising from the growing body of insights into its precise mechanisms—are highlighted.

## Introduction

Atherosclerosis is a chronic disease characterized by the accumulation of lipoprotein particles and inflammatory cells inside the arterial vessel wall of large- and medium-sized arteries. Atherosclerotic plaques may destabilize during the progression of the disease leading to plaque rupture/erosion ultimately resulting in partial or complete vessel obstruction which may cause cardiovascular events such as myocardial infarction (MI) or stroke (1). Altogether, cardiovascular diseases represent the leading cause of death worldwide (2). Mechanistically, atherosclerosis has long been considered a solely metabolic-driven disease; a result of high plasma lipid levels and passive uptake of cholesterol into the vessel wall at atherosclerosis-prone regions marked by disturbed blood flow patterns (3, 4). Over the past decades, however, evidence accumulated highlighting the contribution of immune cells in the etiology of atherosclerosis (4). Leukocytes, the effector cells of the immune system, contribute to all stages of the disease. Specifically, monocyte-derived macrophages, neutrophils and T lymphocytes are involved in inflammatory processes inside the vessel wall during lesion initiation, progression and rupture (5, 6). Thus, leukocyte recruitment to the vessel represents an essential early step in initiation and progression of atherosclerosis preceding local actions of intimal leukocytes. In this review, we aim to summarize basic concepts of leukocyte recruitment and highlight novel findings in the context of atherosclerosis.

## Basic Concepts of Leukocyte Recruitment in Vessels

Leukocyte recruitment from blood to distinct tissues is essential in both non-sterile and sterile inflammation but also under steady-state conditions (7). For simplicity, this review will focus on leukocyte recruitment to diseased vessels in the context of atherosclerosis. Classically, the major players in the recruitment process are the endothelium and different leukocyte subsets including monocytes, neutrophils and lymphocytes. To achieve selective recruitment, all players interact in a strictly orchestrated manner (8). The traditional model of the leukocyte recruitment cascade describes three major steps following tissue/endothelial cell (EC) activation: rolling, activation, and arrest (9). However, recent experimental evidence has expanded our knowledge on the leukocyte recruitment process, suggested additional steps, and refined the molecular principles underlying different stages (10). Within the next paragraph, we aim to outline basic and novel concepts of leukocyte recruitment from the circulation.

### Inflammatory Tissue Activation

Inflammatory tissue activation is the initial step in the leukocyte recruitment cascade in both non-sterile (infectious) and sterile (non-infectious) diseases. It occurs as a physiological response of the immune system to various stimuli including tissue damage and cell death, pathogens, or toxic compounds (11). While some responses are shared between non-sterile and sterile diseases, some are specific to certain pathologies. In this context, atherosclerosis-specific mechanisms will be further outlined in later sections.

Classically, acute inflammation is triggered by conserved pathogen-associated molecular patterns (PAMPs) and endogenous stress signals [damage-associated molecular patterns (DAMPs)] that are recognized by respective receptors on tissue-resident immune cells and non-immune cells (12, 13). Activation of these receptors results in the release of pro-inflammatory cytokines and chemokines (14–16). ECs are one of the main targets of pro-inflammatory cytokines (17, 18). As a result, they upregulate adhesion molecule and chemokine expression. Some cytokines such as histamine activate ECs by binding to G-protein-coupled receptors (GPCRs; = type I activation) and induce intracellular signaling cascades that lead to rapid translocation of preformed molecules (19). In contrast, type II activation of ECs is slower but longer lasting (20). It can be triggered by various inflammatory cytokines, such as tumor necrosis factor alpha (TNFα) and interleukin (IL)1-β, and leads to *de novo* synthesis of adhesion molecules and chemokines (20, 21). The expression and extracellular secretion of various chemokines such as C-C motif chemokine ligand (CCL)2 and C-X-C motif chemokine ligand (CXCL)1 leads to—among other functions—attraction of leukocytes (=chemotaxis) (22–24). Moreover, activated tissue-resident leukocytes, specifically macrophages, can also secrete chemotactic molecules such as CCL3 (23). In addition, activated platelets can deposit chemokines such as CCL5 and CXCL4 on ECs contributing to the chemotaxis of leukocytes to sites of inflammation (25, 26). Of note, differential recruitment of leukocyte subsets is favored by the specificity of certain chemotactic molecules and their respective receptors on leukocytes (27, 28).

In summary, inflammatory tissue activation precedes actual leukocyte recruitment by priming ECs and inducing leukocyte chemotaxis.

### Leukocyte Tethering and (Slow) Rolling

Leukocyte tethering (=capture) and subsequent rolling is the first interaction step between ECs and leukocytes. It is mainly mediated by platelet (P)-selectin, endothelial (E)-selectin and leukocyte (L)-selectin (29). Although first described in platelets, P-selectin is also expressed on activated ECs. Selectins consist of an extracellular N-terminal lectin domain, an epidermal growth factor-like domain, a series of repetitive complement control proteins, a transmembrane domain and a C-terminal intracellular domain (20). With their N-terminal lectin domain, they are able to bind to glycosylated ligands in a calcium-dependent manner on the cell surface of opposite cells (30). Of note, selectin binding is highly dependent on correct glycosylation involving modifications by several enzymes that link various types of saccharide molecules (10, 31).

In vessels, leukocyte rolling is predominantly achieved by the interaction of endothelial E- and P-selectin with P-selectin glycoprotein ligand-1 (PSGL-1) and other glycosylated ligands [e.g., CD44 and ESL-1 (E-selectin ligand 1, specifically binding to E-selectin)] on leukocytes (32–36). In ECs, P-selectin is prestored in vesicles called “Weibel-Palade bodies” and translocated to the luminal membrane as a response to inflammatory stimuli, while E-selectin is synthesized *de novo* upon cell activation (19). L-selectin also interacts with PSGL-1 but is mainly expressed on leukocytes and thus important specifically in secondary leukocyte capture (37, 38). L-selectin on leukocytes was also shown to interact with glycosylated ligands on the endothelial membrane (39–41). However, these experiments were mainly performed in the context of lymph node homing. Still, PSGL-1 is expressed on both leukocytes and vascular ECs (42), which suggests a relevant contribution of L-selectin-mediated rolling in vessels. Additional to leukocyte-leukocyte interactions, platelet-leukocyte interactions are involved in secondary leukocyte capture processes (43, 44). Platelet P-selectin can engage with both endothelial and leukocyte PSGL-1, thereby acting as a bridge between the two cell types. Recent studies have identified further relevant players, such as the interaction of leukocyte macrophage receptor 1 (Mac-1) with platelet CD147 (45) in platelet-mediated leukocyte recruitment. In addition to membrane-bound selectins, extracellular matrix proteins, such as galectins, are likely to be involved in the leukocyte adhesion cascade including rolling (46). Here, extracellular galectins might bind glycosylated ligands on both ECs and leukocytes thus facilitating further interactions. Indeed, slow rolling was impaired in Galectin-3 knockout mice (47). Taken together, leukocyte rolling on ECs is mediated by the interplay of molecules on both cell types. Regarding the important role of endothelial P- and E-selectin, prior EC activation and subsequent selectin expression is key to initiate the leukocyte rolling process.

Recent experimental evidence indicated so-called “integrin-mediated rolling” and “slow rolling” as intermediate steps between rolling and firm arrest (10, 48). Integrin-mediated rolling is achieved by a transient interaction of leukocyte integrins in an intermediate conformational state with their respective adhesion molecules on ECs (10). To some extent, integrin-mediated rolling is also selectin-dependent (49, 50). Although selectins do not bind to leukocyte integrins directly, they can induce integrin activation via an intracellular signaling cascade, for example via leukocyte PSGL-1 (51–53). As a consequence, leukocyte integrins undergo conformational changes that result in increased binding to respective endothelial adhesion molecules (54). Of note, also soluble selectins, known biomarkers for inflammation (55, 56), can induce integrin activation (57). Slow rolling can be viewed as a specific type of integrin-mediated rolling: It is induced by pro-inflammatory cytokine exposure, and mediated mainly by the subsequent upregulation of endothelial E-selectin which induces integrin activation (10, 58).

As described above, myeloid cells, namely monocytes/macrophages and neutrophils, exert key functions in atherosclerosis. Recent evidence suggests that neutrophils are among the first cells recruited to inflamed tissues, thereby facilitating subsequent monocyte uptake (59, 60). This suggests underlying differences in the recruitment cascade of neutrophils and monocytes. Indeed, differences in integrin-mediated rolling and slow rolling have been described. In monocytes, integrin-mediated rolling is mainly conveyed via ß1-integrins such as very late antigen 4 (VLA-4) (10, 61). In contrast, ß2-integrins such as lymphocyte function-associated antigen 1 (LFA-1) and Mac-1 seem to be crucial for integrin-mediated rolling and slow rolling in neutrophils (49, 58, 62).

Taken together, initial leukocyte tethering and subsequent rolling and slow rolling is essential to enable leukocyte contact with ECs.

### Leukocyte Activation and Arrest

Following leukocyte rolling, leukocytes need to firmly adhere to the endothelium for further transmigration. This is achieved by stable interactions between leukocyte integrins and endothelial adhesion molecules (19). However, this process requires prior activation of leukocyte integrins (63). Integrin activation is mainly mediated via so-called “inside-out signaling”; that means the activation of intracellular signaling cascades in response to chemokine binding to dedicated receptors on leukocytes (19). Concretely, secreted chemokines that are present in the extracellular glycocalyx bind to GPCRs on leukocytes, which results in conformational (=affinity) and expression density (=avidity/valency) changes (8, 64). Traditionally, chemokines were supposed to be presented by EC-bound glycosaminoglycans (65, 66). This is contrasted by new experimental studies indicating only transient interactions between chemokines and glycosaminoglycans, which allows retention of chemokines in the glycocalyx space close to the endothelium. However, most likely mainly free chemokines have the ability to bind to GPCRs on leukocytes (67). Chemokines inducing integrin activation are often the same molecules relevant for chemotaxis but quantitative differences in receptor expression and ligand binding may explain the differences in chemoattractant and pro-adhesive response (68).

Upon chemokine binding to GPCRs, complex intracellular signaling cascades get activated which are reviewed in detail elsewhere and are still not deciphered completely (8, 69). One major downstream effect, as mentioned above, are conformational changes in the extracellular domains of leukocyte integrins (from a bent, low-affinity conformation to an extended, high-affinity conformation) (69). Recently, talin and kindlin-3 have been identified as two intracellular molecules that bind to the cytoplasmic tail of integrins and, independent of each other, increase integrin affinity via conformational changes (8, 70–73).

Following activation, heterodimeric leukocyte integrins engage with their counterreceptors on ECs and consequently, leukocytes firmly adhere to the endothelium. Classical integrin-adhesion molecule combinations include interactions between VLA-4 with vascular cell adhesion molecule 1 (VCAM-1), LFA-1 with intercellular adhesion molecule (ICAM)-1, ICAM-2 and ICAM-3, and Mac-1—which is specifically described in human neutrophil arrest (74)—with ICAM-1 (75–79). Additionally, Mac-1 binding to CD40 ligand (CD40L) has been recently identified to contribute to the leukocyte arrest process (80–82).

In contrast to “inside-out signaling,” “outside-in signaling” describes the process in which signals are transduced into leukocytes upon integrin engagement (83). Several studies suggest that this mechanism is specifically important in post-arrest adhesion strengthening and further leukocyte activation (83–85).

Firm arrest is crucial for leukocyte emigration into inflamed tissues as it paves the way for final transmigration through the vascular endothelium.

### Crawling and Leukocyte Transmigration

Compared to the preceding steps of the leukocyte recruitment cascade, detailed understanding of the final step, leukocyte transmigration, has been achieved fairly recently. Intraluminal crawling is essential to later leukocyte transmigration as cells can thereby migrate to preferred sites of transmigration (86). Leukocyte Mac-1 and LFA-1 binding to endothelial ICAM-1 play a key role in the process of crawling (87–90). Crawling and subsequent leukocyte transmigration is promoted by various stimuli (10). In particular, binding of leukocyte integrins to adhesion molecules was shown to induce EC activation (91), comparable to the outside-in-signaling as observed during firm arrest in leukocytes. Concretely, integrin-adhesion molecule interactions result in the clustering of adhesion molecules in specific EC regions yielding ICAM-1- and VCAM-1-rich domains (10, 92, 93).

Leukocyte transmigration can happen via two different routes: the classical, paracellular route (migration between two EC bodies) or a transcellular route (migration through thin parts of an EC body) (89). If migration happens via the paracellular route, EC junctions need to be modified transiently to reduce contact between adjacent ECs. This is achieved, for example, by active squeezing of leukocyte nuclei to disassemble endothelial actin filaments (94), by EC contraction mediated by cytoplasmic structural proteins and by reduced expression of EC adherens junctions such as vascular endothelial cadherin (VE-cadherin) (51, 95). Mechanistically, this is supposed to be triggered by intracellular signaling cascades as a response to LFA-1 and VLA-4 binding and subsequent ICAM-1 and VCAM-1 clustering on EC, which results in increased cytosolic calcium levels leading to enhanced myosin light chain kinase activity, dissociation of vascular endothelial protein tyrosine phosphatase (VE-PTP) from VE-Cadherin and phosphorylation as well as dephosphorylation of distinct tyrosine residues on VE-cadherin (96–98). As a result, VE-cadherin, which is linked to the actin-cytoskeleton via catenins, dissociates from this connection and is internalized, thus contributing to loosening of endothelial tight junctions (99). By contrast, other adherens junction molecules, such as platelet/endothelial cell-adhesion molecule 1 (PECAM-1/CD31) and CD99, but also tight junction molecules, such as junctional adhesion molecule (JAM)-A, are actively transported to the site of diapedesis in so-called lateral border recycling compartments (LBRC) (8, 100). By both homophilic (leukocytes express identical molecules) and heterophilic (leukocytes express non-identical ligands) interactions, endothelial adhesion and junctional proteins achieve shuffling of the migrating leukocyte through the lateral border (91, 101, 102).

A second route using transcellular migration has been described, specifically at thin parts of ECs (93, 103). In this case, tight junctions between ECs remain intact (103). Instead, leukocytes are transported through the EC body by caveolin 1-rich and adhesion molecule-rich (specifically ICAM-1) transcellular pores (95).

However, despite our increasing knowledge on the molecular processes of para- and transcellular leukocyte migration, the mechanisms that decide on the actual migration route have not yet been deciphered completely. The decision is most likely based on a combination of factors including vessel type (macrovascular vs. microvascular), leukocyte subset, tight junctional organization, inflammatory activation and other, yet unidentified aspects (89, 104).

## Leukocyte Recruitment in Atherosclerosis

Whereas the molecular mechanisms of leukocyte recruitment are being elucidated in increasing detail, our knowledge of the specific processes that drive leukocyte migration into atherosclerotic plaques is still rather incomplete. It is likely that there are many shared steps, although some players appear to be more important in atherosclerosis than in recruitment cascades in other tissues. While the involvement of different leukocyte populations in atherosclerosis is known since long on an observational level from histological specimens, most of our current understanding in this field is derived from experiments with induced atherosclerosis in genetically altered mice. Important models for this are mouse lines lacking the genes for apolipoprotein E (*Apoe*) or low density lipoprotein receptor (*Ldlr*) fed a cholesterol-enriched diet (in the following referred to as *Apoe*^−/−^ and *Ldlr*^−/−^ mice, respectively).

**Figure 1:**
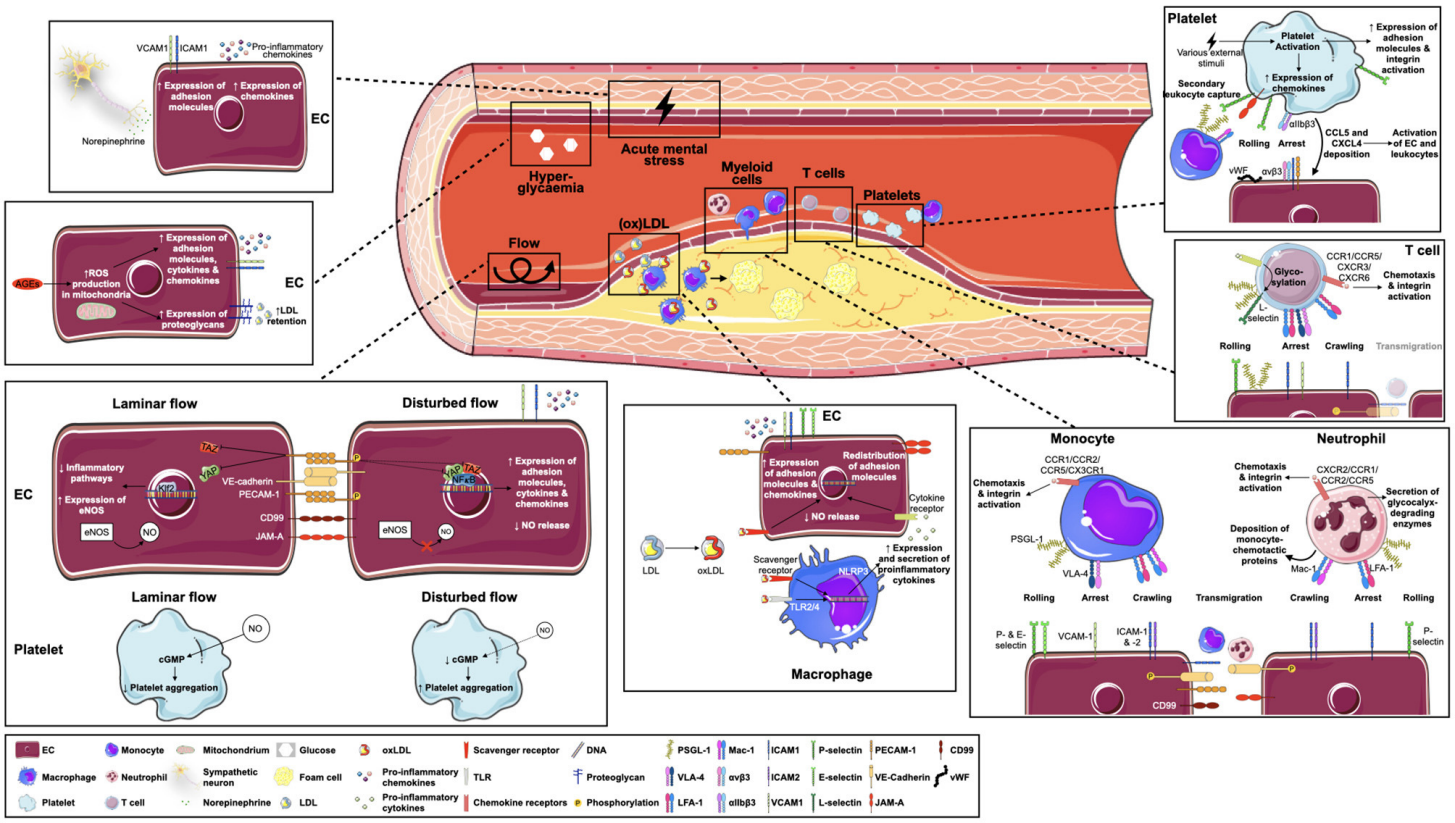
Key factors in atherosclerosis-specific leukocyte recruitment. Leukocyte recruitment into atherosclerotic plaques is multifaceted and involves several players. Endothelial activation through disturbed flow patterns and oxidized lipoproteins, but possibly also via hyperglycemia or local sympathetic innervation, forms the basis for subsequent leukocyte-endothelial interactions. Of note, specific leukocyte populations respond differently to atherogenic triggers and use unique molecules and receptors to achieve leukocyte rolling, arrest, crawling and transmigration. Additionally, platelets strongly contribute to leukocyte recruitment by secondary leukocyte capture and activation of endothelial cells and leukocytes. AEG = advanced glycosylation end products; CCL, C-C motif chemokine ligand; CCR, C-C motif chemokine receptor; CD99, cluster of differentiation 99; cGMP, cyclic guanosine monophosphate; CXCL, C-X-C motif chemokine ligand; CX(3)CR, C-X(3)-C motif chemokine receptor; EC, endothelial cell; eNOS, endothelial nitric oxide synthase; E-selectin, endothelial selectin; ICAM-1/2, intracellular adhesion molecule 1/2; Klf2, Krüppel-like factor 2; NLRP3, NOD-, LRR- and pyrin domain-containing protein 3; L-selectin, leukocyte selectin; Mac-1, macrophage receptor 1; NO, nitric oxide; (ox)LDL, (oxidized) lipoprotein; PECAM-1, platelet endothelial cell adhesion molecule 1; P-selectin, platelet selectin; PSGL-1, P-selectin glycoprotein ligand 1; ROS, reactive oxygen species; TLR, toll-like receptor; VCAM-1, vascular adhesion molecule 1; VE-cadherin, vascular endothelial cadherin; VLA-4, very late antigen 4; vWF, von Willebrand factor, YAP, yes-associated protein.

Increasingly, these findings may also be supported by the results of genetic or proteomic association studies in humans: Single nucleotide polymorphisms (SNPs), for example, are used to compare the individual genetic profile of coronary artery disease (CAD) patients and healthy controls, and the enrichment of certain SNPs in the patient cohort can accordingly be linked to CAD in so-called genome-wide association studies (GWAS). Thus, with increasing numbers of SNPs and individuals included, a large number of variants associated with CAD have already been identified (105, 106). Although many of these SNPs reside in areas of unknown function in the genome, some of the associated genes have already been linked to leukocyte recruitment processes (107). Bringing together knowledge derived from these different approaches, we aim to summarize and discuss the currently known important players in immune cell recruitment into atherosclerotic plaques below (Figure 1).

### Endothelial Cell Priming

#### Keep It Flowing

The endothelium is permanently exposed to blood flow-induced shear stress and adapts to changes in flow by several immediate responses, e.g., conformational remodeling of the glycocalyx, opening of ion channels, and activation of different membrane receptors such as GPCRs and integrins (108). A central role in these mechanotransduced responses can be attributed to the mechanosensory PECAM-1, VE-cadherin and vascular endothelial growth factor receptor (VEGFR) complex: Acute onset of laminar flow promotes PECAM-1 phosphorylation followed by Src-dependent phosphorylation of VEGFR-2 and−3, proteins which are both linked to PECAM-1 via VE-cadherin and subsequently activate multiple intracellular pathways (109–111). Many downstream functions of this complex are transmitted via phosphatidylinositol 3-kinase/Akt signaling, e.g., leading to global activation of β1-integrins and the small GTPase RhoA which finally triggers promotion of focal adhesions, cytoskeletal adaption and alignment of the cell in the direction of flow (112, 113), or activation of endothelial nitric oxide (NO) synthase (eNOS) leading to NO-mediated vasodilation (114–116). Lastly, these processes stimulate the activation of transcription factors such as Krüppel-like factor 2 (Klf2) (117) and inhibit pro-inflammatory action of the Hippo pathway effectors yes-associated protein (YAP) and transcriptional coactivator with PDZ-binding motif (TAZ), which have been identified as key mechanotransducers in response to disturbed flow (118). In summary, initial pro-inflammatory activity triggered by flow onset is followed by alignment of the cell in the direction of flow, enhanced NO production and inhibition of inflammatory pathways, maintaining an anti-inflammatory status of the endothelium in response to laminar shear.

In contrast, disturbed flow, which preferentially occurs in branches, bifurcations and curvatures of the vessel—regions, where atherosclerotic plaques are mainly found—has been shown to lead to different responses: impaired NO release (119, 120), reactive oxygen species (ROS) production (121), deposition of fibronectin and fibrinogen to the subendothelial basement membrane (122, 123), and activation of pro-inflammatory transcription factors such as nuclear factor (NF)-κB and the aforementioned transcription cofactors YAP/TAZ, inducing the expression of several pro-inflammatory proteins such as VCAM-1, ICAM-1, CCL2, IL-6, and CXCL8 (111, 118, 124). Additionally, shear stress response regulatory elements have been found in the promoter of *NOS3*—the gene encoding eNOS—downregulating its expression in response to flow (125). Apart from that, evidence also suggests a flow-dependent expression of Toll-like receptor (TLR) 2 on EC (126, 127). Therefore, permanent changes in shear stress hinder the cell from adapting to the direction of flow and from overcoming the initial pro-inflammatory response phase, rendering these areas prone to leukocyte influx. This is also mirrored by the role of PECAM-1: While loss of *PECAM1* is associated with reduced NO production in EC exposed to laminar flow (115, 128) and was shown to accelerate the onset of collagen-induced arthritis in mice (129, 130), in regions of disturbed flow, PECAM-1 elicits pro-inflammatory effects in the vasculature via increased NF-κB activation and VCAM-1 expression as shown in *Pecam1*-knockout mouse models (111, 131). Accordingly, more recent findings led to the conclusion that PECAM-1 exerts both pro- and anti-atherosclerotic properties in EC depending on the type of flow (132, 133). Moreover, common variants assigned to *PECAM1* in humans have been associated with CAD in a large GWAS (134), highlighting the role of PECAM-1 in atherosclerosis beyond its known effects in cell culture and mouse models.

Another striking role in the abovementioned process of flow-induced EC priming can be attributed to NO, a gas produced mainly by eNOS that diffuses across cell membranes and, via soluble guanylyl cyclase (sGC), promotes the formation of the second messenger cyclic guanosine monophosphate (cGMP) in various neighboring cells. Among its several important downstream functions are smooth muscle cell relaxation, thus regulating the vascular tone, and platelet aggregation (135). Recently, it was shown that not only NO production in EC but also cGMP formation in one of its effector cells, namely platelets, is strongly shear-dependent (136). Insufficient NO availability, as it may be caused by impaired blood flow, is the major cause of endothelial dysfunction and leads to several pro-atherogenic responses in the vasculature by directly impacting leukocyte recruitment, e.g., increasing NF-κB activation and enhancing endothelial expression of VCAM-1, E-Selectin, and ICAM-1 (137). Furthermore, common polymorphisms in *NOS3*, but strikingly also several other genes of this pathway—among them *GUCY1A1* encoding for the α_1_-subunit of sGC and *PDE5A* for a cGMP degrading enzyme—have been linked to coronary artery disease susceptibility in GWAS (138–140) and once again highlight the role of this pathway in atherogenesis.

#### oxLDL and Other Evildoers

The second important pillar for atherosclerotic EC priming is attributed to inflammatory mediators. While the role of PAMPs in atherogenesis is not investigated to a large extent, although the role of viruses and bacteria on plaque progression or rupture has been increasingly recognized (141), most research refers to the sterile character of atherosclerotic inflammation. In this context, modified low density lipoprotein (LDL) plays a fundamental role.

LDL from the circulation can be incorporated by EC either by receptor-mediated endocytosis or caveolae mediated transcytosis (142, 143), whereby the latter one is generally regarded as the more relevant way of atherogenic LDL accumulation (144). In the subendothelial space, LDL is retained by extracellular matrix proteoglycans (145) and, catalyzed by enzymes such as lipoxygenases or myeloperoxidases, metal ions and free radicals, chemically modified to various degrees. This leads to the formation of strongly pro-atherogenic LDL variants such as oxidized LDL (oxLDL) (137). The exact process of LDL oxidation is not fully elucidated but is supposed to be linked to oxidative stress resulting from a disbalance between ROS production and antioxidant defense mechanisms (146), as triggered, for example, by aging (147) or smoking (148). As a consequence, LDL loses its ability to bind the LDL receptor but strongly enhances affinity for scavenger receptors such as CD36 and lectin-like oxidized LDL receptor-1 (LOX-1) on EC, vascular smooth muscle cells, and macrophages (149). Intriguingly, a degradation product of LOX-1 can also be found in plasma [soluble Lox-1 (sLox-1)] and has recently emerged as a potential biomarker for cardiovascular disease incidence (150).

Ultimately, among the myriad pro-atherogenic responses of EC to such modified LDL are inhibition of NO production (151, 152), regulation of microRNAs (153), enhanced expression of E- and P-selectin, VCAM-1, ICAM-1 (154, 155), CCL2, CXCL2, 3 and 8 (156), and redistribution of JAM-A to facilitate transmigration (157)—thus paving the way for leukocyte infiltration. Therefore, oxidation of LDL is without doubt a central aspect of atherogenesis (149). However, while oxLDL is the best-studied form of modified LDL, there are several other modifications of LDL with pro-atherogenic properties, such as desialylation (158) or sphingomyosinase-induced aggregation (159).

Strikingly, these processes appear to begin already very early in life in genetically predisposed individuals (160), highlighting the influence of heritable factors on atherogenesis, most of which remain unexplored (161). Among the known contributors to atherogenic EC priming, however, is also hyperglycaemia. For example, hyperglycaemia driven accumulation of advanced glycosylation end products (AGEs) in vessels promotes ROS formation and adhesion molecule expression in EC, and release of pro-inflammatory cytokines such as IL-1β, IL-6, CCL2 and CXCL8 from leukocytes (162). Moreover, it impairs eNOS function and promotes the expression of proteoglycans, associated with increased LDL retention in the vascular wall (137, 163). Another important driver of atherogenesis is psychological stress (164, 165). Just recently, we showed that acute mental stress promotes atherosclerosis-related recruitment of leukocytes in mice by increasing the expression of endothelial adhesion molecules and the release of chemokines (166), adding to the knowledge of neuroimmune linkages in atherosclerosis.

### Stage Free for Leukocytes

In response to upregulated adhesion molecules on EC and an increased chemokine gradient, leukocyte adhesion is initiated. However, this is not a static “one after the other” process. Several inflammatory stimuli emanating from vascular cells, platelets and leukocytes—as a response to the flow and lipid-driven EC priming, but also interlocking from the very beginning—continuously contribute to EC activation. Therefore, this section will focus on the role of the second important player in the atherosclerotic recruitment process: leukocytes.

#### The Classic: Monocytes

Monocytes and macrophages are the central figures in the history of atherosclerosis research. Once migrated, monocytes differentiate into macrophages, the major leukocyte population within atherosclerotic plaques (167), which strongly engulf modified LDL and fulfill several proatherogenic functions. There are three main subsets of monocyte populations in humans, that is classical CD14^++^ CD16^−^ and non-classical CD14^+^ CD16^++^ monocytes and a small, less investigated group of intermediate CD14^+^ CD16^+^ monocytes (168, 169). In mice, C-C motif chemokine receptor (CCR)2^+^ C-X3-C motif chemokine receptor (CX3CR)1^+^ lymphocyte antigen 6 complex, locus C (Ly6C)^high^ monocytes are closely related to the classical cells in humans and CCR2^−^ CX3CR1^++^ Ly6C^low^ monocytes to the non-classical population (170). Although fate mapping and adoptive transfer experiments suggest that Ly6C^low^ monocytes are derived from Ly6C^high^ cells, hypercholesterolaemia is associated with impaired Ly6C^low^ formation, despite a strong expansion of the Ly6C^high^ population leading to systemic monocytosis (171–173). Importantly, both monocyte subsets fulfill different roles in monocyte recruitment. In steady state, Ly6C^low^ monocytes are dependent on CX3CL1 (fractalkine) stimulation via CX3CR1 and are constantly patrolling the vessel by communicating with endothelial ICAM-1 and−2 via LFA-1, but scarcely transmigrate (174). Although in the setting of tissue damage it has been suggested that they are among the first cells to extravasate and to promote recruitment of other leukocytes by release of inflammatory cytokines (175), Ly6C^low^ monocytes were associated with markedly less recruitment to atherosclerotic plaques than Ly6C^high^ monocytes (176). However, under atherosclerotic conditions their patrolling behavior is strongly upregulated in a CX3CR1-independent manner, and genetic depletion of Ly6C^low^ monocytes was associated with pronounced endothelial apoptosis, suggesting an important role for endothelial maintenance in atherosclerosis (177). Nonetheless, in the following, we focus on the recruitment of inflammatory monocytes.

An initial trigger for leukocyte extravasation are local chemotactic gradients. For monocytes, CCL2 is a key chemokine that targets the CCR2 receptor highly expressed on classical/ Ly6C^high^ monocytes (28). Both *Ccr2* and *Ccl2* knockout in atherosclerosis-prone mice (178, 179) as well as *Ccr2* targeted siRNA treatment (180) significantly reduced atherosclerotic plaque formation, whereas in contrast, leukocyte-specific overexpression of *Ccl2* in *Apoe*^−/−^ mice promoted atherosclerosis progression (181). In humans, CCL2 levels in atherosclerotic lesions have only recently emerged as a potential indicator of plaque vulnerability (182). Interestingly, individuals with familiar hypercholesterolemia—a strong genetic predisposition to atherosclerosis—were found to have a 3-fold higher CCR2 expression on classical monocytes than healthy subjects, whereas cholesterol lowering therapy with a proprotein convertase subtilisin/kexin type 9 (PCSK9) antibody reduced monocyte CCR2 surface expression by 60% in these patients (183). This is further evidence that lipid-related and inflammatory processes strongly interact. Yet, despite the striking role of CCR2, also CCR5 is crucially involved in Ly6C^high^ monocyte chemotaxis. While *Ccr5* deficiency resulted in reduced mononuclear cell infiltration and lesion formation as well as decreased neointima formation (184–186), combined inhibition of CCL2, CX3CR1, and CCR5 resulted in as much as a 90 % reduction in atherosclerotic plaque formation in *Apoe*^−/−^ mice, in spite of persistent hypercholesterolaemia (187). However, rather than having chemotactic functions, the CX3CR1/CX3CL1 interaction may impact cell survival (188).

Following attraction to atherosclerosis-prone endothelium, pro-inflammatory monocytes initiate rolling particularly by interaction of PSGL-1 with P- and E-selectin expressed on activated EC. *Apoe*^−/−^ mice with a functional knockout of the gene encoding for PSGL-1 were shown to develop smaller atherosclerotic plaques (189) and in a similar way, P-selectin deficiency was associated with less leukocyte recruitment in atherosclerosis (190–192). Plaque leukocyte recruitment and consequently plaque size were also decreased when atherosclerotic mice were treated with EC-avid nanoparticles inducing endothelial silencing of P- and E-selectin, in parallel with ICAM-1, ICAM-2 and VCAM-1 (193). While lack of E-selectin alone reduced the progression of atherosclerotic plaque formation only to a minor extent (194), the combined genetic silencing of P-and E-selectin in *Ldlr*^−/−^ mice even led to an 80% reduction in lesion formation (195). An inhibitory peptide preventing monocyte binding to selectins was shown to decrease monocyte recruitment and subsequently atherosclerotic lesion size, particularly by inhibiting monocyte activation via NF-κB (196). Moreover, intravital microscopy experiments in inflamed cremasteric veins indicated that E-selectin selectively affects the rolling velocity of inflammatory monocytes, whereas the flux of rolling neutrophils is regulated by P- and L-selectin (197). However, it is open whether this is also true for atherosclerotic arteries. As described above, transition of rolling to slow rolling and eventually to firm arrest requires integrin activation on the surface of leukocytes (102). Intravital microscopy in carotid arteries revealed a crucial function of CCR1 and CCR5 in this step for classical monocytes, but not of CCR2 (198). Although monocytes, in common with neutrophils, express the integrins LFA-1 and Mac-1, monocyte arrest seems to depend particularly on the VLA-4-VCAM-1 interaction (199–201). In line, blocking VLA-4 decreased leukocyte recruitment in atherosclerotic mice (202, 203) and functional downregulation of *Vcam1* significantly reduced atherosclerotic lesion formation in mice in a gene-dose dependent manner (204, 205), while similarly, treatment with a VCAM-1 blocking antibody attenuated atherosclerosis in *Apoe*^−/−^ mice (206). Of note, it has been shown that rolling of monocytes may also be mediated by platelets bound to the extracellular membrane, but rather in a ß2-integrin dependent way (207).

When firmly attached to the endothelium, monocytes engage in crawling behavior in search of ideal sites for extravasation. Thereby, using the intracellular actomyosin machinery for directed movement, they develop integrin-rich protrusions that scan the endothelial lumen for chemotactic directionality (102) mainly by interacting with ICAM-1 or ICAM-2 on EC via both LFA-1 and Mac-1 (208). Experiments in mice could partly confirm the involvement of these adhesion molecules in atherosclerosis: *Apoe*^−/−^ *Icam1*^−/−^ mice displayed reduced atherosclerotic lesions (209) and similarly, fatty streak lesion area was smaller in mice deficient for either ICAM-1 or ß2-integrin (the common subunit of LFA-1 and Mac-1), but most distinctly for mice with a double knockout of both encoding genes (210). Also, *Icam1*^−/−^ *Apoe*^−/−^ mice were shown to have significantly reduced atherosclerotic lesions, and soluble levels of ICAM-1 paralleled atherosclerosis progression in *Apoe*^−/−^ mice with significantly elevated plasma concentrations compared to experiment onset (211). However, other studies could not confirm a substantial involvement of ICAM-1 loss on atherogenesis (204, 212). While the participation of LFA-1, on the other hand, was associated with atherosclerosis progression in rats (213), a study in *Ldlr*^−/−^ mice could not endorse significant influence of Mac-1 on atherosclerotic lesion formation (214). Interestingly, a recent publication proposes a divergent influence of ß2-integrin on different stages of atherosclerosis—being protective in the initial phase, but pro-atherogenic in later stages, which could be partly driven by chronic dyslipidaemia (215).

Transmigration, the final step of the recruitment cascade, is facilitated by the redistribution of adhesion molecules such as JAM-A and VE-cadherin and formation of transmigratory cups characterized by local clustering of ICAM-1 and VCAM-1, actin remodeling and the formation of endothelial protrusions developing around the penetrating leukocyte (86). Thereby, also several effectors of the Rho-GTPase family are activated in EC, such as triple functional domain protein (Trio), Ras-related C3 botulinum toxin substrate 1 (Rac1), RhoG and its exchange factor SH3-containing guanine nucleotide exchange factor (SGEF) (216), stimulating the formation of the cup-like structures during transmigration of leukocytes but also promoting ROS production and subsequent activation of matrix metalloproteinases (MMP). In a later step, also RhoA and its effector Rho-associated protein kinase (ROCK) are activated, enlarging the transmigratory gap through enhanced actin-myosin contractility (217). Indeed, SGEF-deficient mice displayed decreased atherosclerotic plaque formation supposedly via reduced formation of endothelial docking structures (218) and similarly, inhibition of ROCK reduces atherosclerosis in mice (219, 220). Strikingly, the genes encoding for RhoA, Rac1 and SGEF were also associated with CAD by GWAS (139, 221–223).

Further, VE-cadherin plays a crucial role in leukocyte transmigration. It is linked to the actin-cytoskeleton via catenins and constitutively associated with the phosphatase VE-PTP, which stabilizes VE-cadherin junctions both by dephosphorylation and inhibition of Rho GTPase signaling (96, 224, 225). Beyond its previously described effects, oxLDL was also shown to directly promote monocyte transmigration by down-regulating VE-cadherin and upregulating PECAM-1 (226). Besides, PECAM-1 deficiency or blocking antibodies have been shown to specifically inhibit transmigration *in vitro* and *in vivo* in various inflammatory disease models in mice (99). Together with CD99, but also JAM-A, PECAM-1 is actively transported to the transmigration site via LBRCs and likely required for leukocyte diapedesis, as blocking of this targeted process resulted in considerably lower monocyte transmigration *in vitro* (227). In line, impaired JAM-A expression in EC or blocking JAM-A by a peptide antagonist inhibited leukocyte recruitment and atherosclerotic plaque formation in hyperlipidemic mice (228, 229). Remarkably, also the expression and distribution of JAM-A appears to respond to changes in flow (230). Given that CD99 also exerts an important influence on monocyte transmigration (231), in an interesting experiment, vaccination directed against CD99 was shown to reduce leukocyte numbers in atherosclerotic plaques and attenuate atherosclerotic lesion formation in mice (232). However, the role of PECAM-1—although quite obviously promoting leukocyte transmigration *in vitro*—seems to be more complex in atherosclerosis in general, as it exerts different influences on atherogenesis partly depending on the hemodynamic environment (see section Keep it Flowing).

Following their recruitment into plaques, monocytes massively differentiate into macrophages and, to a lesser extent, presumably also into dendritic cells (233). However, accumulation of macrophages in atherosclerotic lesions is likely a complex interplay of monocyte recruitment and local macrophage proliferation which also involves tissue-resident macrophages (234, 235). Within plaques, macrophages strongly engulf modified LDL particularly via scavenger receptor A1 (SRA1), LOX-1 and CD36, which is supported by TLR2, 4 and 6 signaling and promoting their phenotypic change to cholesterol-rich foam cells (236). Subsequently, foam cells can induce the release of pro-inflammatory cytokines (237) and *Vcam1* expression in early aortic fatty streaks in mice (238). Excessive cholesterol accumulation may also lead to the formation of cholesterol crystals which trigger activation of the NOD-, LRR- and pyrin domain-containing protein 3 (NLRP3) inflammasome, a huge cytosolic oligomer inducing cleavage and secretion of IL-1ß and IL-18 (239). However, the inflammatory role of foam cells has been challenged by recent findings, which support a more diverse function in atherosclerosis (240) and suggest non-foamy macrophages to be the actual contributors of pro-inflammatory signaling in atherosclerotic plaques (241). As such, monocyte-derived macrophages again strongly promote endothelial activation and subsequent invasion of additional leukocytes, so permitting the plaque to grow and grow under continuous LDL supply (242).

#### The Newcomer: Neutrophils

Neutrophils are the most abundant leukocyte population in human blood and it has been just in the course of the last decades that they emerged from more or less neglected bystanders in atherogenesis to forerunners of monocyte infiltration (243). In infection, they are among the first cells to invade into inflamed tissues and promptly release cytotoxic ROS and proteases or form neutrophil extracellular traps (NET) targeted to rapidly eliminate pathogens (77). However, such behavior has also been observed in sterile inflammation as in atherosclerosis, making neutrophils furthermore an important contributor to atherosclerosis progression and complications such as stroke or acute coronary syndrome (244). While many steps of neutrophil recruitment into inflamed tissues are shared with monocytes and therefore close to the mechanisms described above, below, we focus on specific differences in the recruitment behavior of neutrophils.

Comparable to atherosclerotic monocytosis, also neutrophil levels in the blood are frequently increased in atherosclerosis (245) and related to future major adverse cardiovascular events in patients with acute coronary syndrome (246). Neutrophils circulating in the bloodstream are highly sensitive to various chemotactic signals. The traditional view that monocyte chemotaxis rather depends on CC-chemokines and neutrophil chemotaxis rather on CXC-chemokines is supported by predominant expression of CXC-chemokine receptors in neutrophils (247, 248) and fueled by a recent study concluding that CCR1, CCR2, CCR3, and CCR5 are not involved in neutrophil recruitment in acute inflammation in mice (28). However, several other studies have also demonstrated an important role for CC-chemokines in neutrophil attraction (249), suggesting that a strict separation of monocyte and neutrophil relevant chemokines does likely not represent the whole picture. As follows, important chemokines to trigger neutrophil activation in mice are CXCL1, CXCL2, and CXCL5 (presumably also representing CXCL8 in humans) as well as CCL5. Evasin-3, a pharmacological inhibitor of CXCL1 and CXCL2, reduced intraplaque neutrophil and MMP9 content (250). Similarly, the nicotinamide phosphoribosyltransferase inhibitor FK866, which was shown to strongly inhibit CXCL1 production in EC *in vitro*, reduced neutrophil infiltration and MMP-9 content in atherosclerotic lesions (251). Moreover, neutrophil recruitment to large arteries was shown to depend on CCR1, CCR2, CCR5, and C-X-C motif chemokine receptor (CXCR)2 in early stages of atherosclerosis, thus being particularly dependent on platelet-derived CCL5 stimulation (243). Another important role in neutrophil chemotaxis was also shown for CCL3, as leukocyte-specific CCL3 depletion inhibited atherosclerotic lesion formation particularly by affecting neutrophil accumulation (252).

Importantly, neutrophils also contribute to monocyte recruitment by depositing chemotactic proteins on the endothelium (244). One such example is cathelicidin. Mice lacking the corresponding gene developed significantly smaller atherosclerotic lesions with lower numbers of plaque macrophages (253). Similarly, it was reported that neutrophil-derived α-defensin can complex with CCL5 and be presented on EC, causing enhanced monocyte adhesion and vascular inflammation (254); while cathepsin G—also released from granules of neutrophils—is specifically deposited on the arterial endothelium of arteries but not on venule EC, promoting adhesion and extravasation of myeloid cells specifically in atherosclerosis-susceptible areas of vessels (255).

Neutrophils can facilitate their way to the endothelium through the dense network of endothelial glycocalyx by releasing proteolytic proteins, MMPs and ROS, thus locally breaking this physical barrier down (256). At the endothelium, neutrophil tethering and rolling is mainly mediated by PSGL-1 binding to P-selectin and CD44, but not to E-selectin, which is thus assumed to be selectively used by inflammatory monocytes (197). This is an important observation, as preformed P-selectin is available much quicker upon activation of EC than E-selectin which requires *de novo* synthesis, and which could therefore partly explain the delayed secondary recruitment of inflammatory monocytes compared to neutrophils in inflammation. CD44 has been studied quite extensively for its involvement in atherosclerosis, but depletion in atherosclerotic mice tended to yield conflicting results (257). Depletion of L-selectin, which might be specifically important in secondary capture, was consensually shown to promote atherosclerosis, accompanied by a drop of aortic B cells (258, 259). *In vivo*, however, the rolling behavior of monocytes and neutrophils on carotid artery bifurcations of *Apoe*^−/−^ mice appears to be quite different: While the number of rolling neutrophils, in contrast to monocytes, increased during high-fat diet, the rolling rate of monocytes decreased during the same period (260). Another striking observation is that under high shear stress, by cytoskeletal reorganization during rolling neutrophils can form slings out of their membrane which are characterized by surface expression of distinct sticky PSGL-1 clusters and LFA-1, thus facilitating contact with the endothelium (261). During rolling, neutrophils were also shown to secrete S100 calcium-binding protein (S100)A8 and S100A9, calcium-binding proteins constitutively expressed in myeloid cells that account for ~45% of the cytoplasmic proteins in neutrophils (262). Interestingly, apart from their numerous functions in inflammation, these proteins were also associated with leukocyte chemotaxis, inducing VCAM-1 and ICAM-1 expression in EC while upregulating Mac-1 expression in leukocytes, subsequently resulting in increased TLR4-mediated Mac-1/ICAM-1 binding, decelerated leukocyte rolling and enhanced firm adhesion on the endothelium (263, 264). In line, blocking antibodies or genetic depletion of S100A9 reduced leukocyte recruitment in several murine inflammatory disease models (262), making these proteins a promising pharmacological target in inflammation-related diseases.

Transition from slow rolling to neutrophil arrest seems to particularly depend on LFA-1 binding to endothelial ICAM-1, which is in contrast to monocytes requiring VLA-4/VCAM-1 interactions. Hereby, CXCR2 is assumed to be of central importance for “inside-out” activation of LFA-1 (265). And while monocyte crawling is dependent on both Mac-1 and LFA-1, neutrophils seem to exclusively crawl via Mac-1, interacting with ICAM-1 and ICAM-2 (88, 208, 266). Remarkably, when Mac-1 is blocked, neutrophils can also crawl against the direction of flow *in vitro* by engaging LFA-1, while Mac-1 favors flow-directed crawling behavior (87). During crawling, neutrophils flatten and form protrusions reaching into the endothelial surface similar to monocytes, while on the front of the moving cell, filamentous actin (F-actin) and on the end, the so-called uropod, myosin filaments aid the cell in directed movement on the endothelium (267). Lack of the actin cytoskeleton transcription factor MKL1 in neutrophils almost completely abrogated migration *in vitro* (268). Importantly, neutrophils were recently found to actively scan for activated platelets via protruding PSGL-1 clusters at the uropod, and such interactions with platelets were crucial for intravascular migration of neutrophils (269). Also EC derived CXCL1 was shown to support neutrophil crawling, while CXCL2, mainly produced by neutrophils themselves, particularly aided in breaching of endothelial junctions together with its atypical chemokine receptor 1 which was found to be enriched in endothelial junctions (270).

Following firm arrest, neutrophil interaction with EC via β2-integrin/ICAM-1 triggers VE-cadherin phosphorylation and subsequent loosening of endothelial adherent junctions to promote the favored paracellular route for diapedesis (97). This is quite similar to the process described for monocytes above, and requires the formation of transmigratory cups which were reported to form around neutrophils specifically in a “dome” shaped manner (271), involving RhoA and leukocyte-specific protein 1 mediated formation of contractile F-actin structures that tightly surround the invading cell in order to prevent vascular leakage during transmigration (272, 273). Strikingly, neutrophils were also observed to return to the circulation by reverse transendothelial migration in the context of low JAM-C expression in mice (274). Mechanistically, local proteolytic cleavage of JAM-C, driven by neutrophil-derived elastase, was shown to promote this behavior (275).

After infiltrating the plaque, neutrophils have a plethora of possibilities to further promote leukocyte recruitment and subsequent progression of atherosclerosis. They can release granula proteins such as cathelicidin, cathepsin G, elastases, MMPs and ROS, or activate NET formation, thus attracting further leukocytes, promoting oxidative stress, LDL modification, EC activation, activation of macrophages and cellular damage (244).

#### The Player With the Many Faces: T Cells

While myeloid cells form the first line of defense and respond rapidly but uniformly to a broad spectrum of identified threats, cells of the adaptive immune system perform highly specific tasks that are individually tailored to the particular profile of their target, resulting in a delayed but finely matched immune response. Thus, it is not surprising that T cells—a highly abundant population in human atherosclerotic plaques—are divided into multiple different subpopulations. This includes naïve, memory and effector CD4+ and CD8+ T cells, but also regulatory T cells (Treg) (276). CD4+ T cells are generally associated with increased atherosclerotic plaque growth (277–280). Following antigen-presentation, CD4+ T cells are activated and differentiate into T helper (Th)1, Th2, Th17 cells or other subsets and resemble, together with memory T cells, the major proportion of T cells to be found within atherosclerotic plaques (281). However, they can also give rise to Tregs in the periphery (282). Among the CD4+ T cells, Th1 cells are the most abundant T-subtype in human atherosclerotic lesions (283) and can generally be attributed as pro-atherosclerotic (284, 285), while the role of the other T cell subsets is still a matter of debate and discussed in detail elsewhere (286). Tregs, on the other hand, which express the characteristic transcription factor forkhead box protein P3 (FOXP3) and CD25, are clearly associated with an anti-inflammatory role by suppressing the proliferation of pro-inflammatory effector T cells and influencing macrophage function toward an anti-inflammatory phenotype (287, 288). Depletion of Treg cells in mice aggravates atherosclerosis (289–291) and Tregs express IL-10 and transforming growth factor (TGF)-β, both associated with anti-atherosclerotic effects in atherosclerosis (292). Interestingly, however, just recently an autoreactive phenotype in Tregs directed against apolipoprotein B-100 (ApoB-100), the core protein of LDL, in late-stage atherosclerosis was identified (293), questioning the classical view of Tregs as solely beneficial cells. Moreover, a recent study showed that dyslipidaemia reprograms the metabolic footprint of Tregs toward an effector-like migratory phenotype, challenging the classical hypothesis that Treg migration into plaques might be reduced (294). Finally, CD8+ T cells are more frequent in blood of CAD patients than in healthy individuals (295, 296) and generally associated with pro-atherosclerotic effects in preclinical studies (297–299), but, in contrast, inhibiting CD8+ cells in advanced lesions also resulted in less stable lesions (300).

T cells can use both classical myeloid cell like and antigen-dependent patterns for migration into tissues (256). While most of the T cells found within plaques are antigen-experienced T cells (286) and T cells targeting ApoB-100 were shown to circulate in human blood (301), a recent study suggests that naïve T cells can also be primed directly in the vessel wall (302). Of note, this was not related to tertiary lymphoid organs which can be found in later stages of atherosclerosis within the adventitia and promote Treg expansion (303). However, the antigens to which T cells respond in atherosclerosis are mostly unknown which renders it difficult to study antigen-dependent effects in T cell migration. Therefore, the precise mechanism of T cell recruitment to atherosclerotic plaques, albeit of great interest, is still subject of basic research, and many of the subsequent insights are derived from *in vitro* findings.

To migrate into murine atherosclerosis-prone vessels, circulating T cells roll on endothelial P-selectin using PSGL-1 *in vivo* (302), with a potential role for its co-factors CD44 and CD43 (304, 305). In contrast to monocytes, however, to be fully active, PSGL-1 in T cells requires prior glycosylation (35). Mac-1 has not been described to participate in T cell recruitment but L-selectin, which is important for lymphocyte trafficking into lymph nodes (256), was shown to play a role in T cell migration into peripheral tissues, especially in recruitment into the adventitia of healthy, non-inflamed arteries in mice (306). This again suggests a role for naïve T cell recruitment, as T cells generally lose L-selectin expression upon antigen-presentation. Notably, in a study using intravital microscopy, rolling of T cells on carotid artery bifurcations could not be observed in early atherosclerosis, but was induced on pronounced atherosclerotic lesions in mice (260), indicating a more pronounced role for T cells in late atherosclerosis.

Induction of firm adhesion requires integrin activation, which is accomplished by chemokines in a similar way as in myeloid cells. Different T cell subsets react to different cytokines, but CCR1 and CCR5 have been shown to be expressed on most atherosclerosis relevant T cells and thus are supposed to play a major role in T cell recruitment in response to CCL5 (307). However, CCR1 and CCR5 appear to exhibit opposing effects, as CCR1 seems to be anti-atherosclerotic in the context of T cell recruitment in murine atherosclerosis (308), whereas CCR5 rather has a pro-atherogenic role (section The Classic: Monocytes). Another chemokine receptor to be found on Th1 cells is CXCR3, which is required for Th1 differentiation (309). It requires binding of CXCL10, which, when inhibited, decreases atherosclerotic lesion size and specifically T cell accumulation in murine atherosclerotic lesions (310). Interestingly, CXCL10 levels were suggested to be higher in obese compared to non-obese subjects, functionally promoting adhesion capacity of leukocytes *in vitro* (311). Similarly, genetic depletion of *Cxcr3* or antagonizing CXCR3 pharmacologically in mice reduced atherosclerosis progression and infiltration of inflammatory T cells, while Treg numbers rised (312, 313). CXCR6 has been described as a marker of polarized Th1 cells (314) important for T cell homing, as absence of CXCR6 inhibited recruitment of T cells, diminished IFNγ production and atherosclerotic lesion formation in *Apoe*^−/−^ mice (315). CXCL16 is the ligand for CXCR6 and chemoattractive when expressed and deposited on EC, but also functions as a scavenger receptor for oxLDL on monocytes and macrophages (316). In contrast to other oxLDL scavenger receptors, CXCL16 depletion was associated with reduced plaque formation in *Ldlr*^−/−^ mice (317), thus acting in both pro- and anti-atherosclerotic ways, depending on the context. Last but not least, CCR7, which mediates T cell homing to lymph nodes, and its ligands CCL19 and CCL21 have also been identified within atherosclerotic lesions of humans and mice (318). However, studies on atherosclerosis in *Ccr7*^−/−^ mice achieved controversial findings (281).

To summarize, several chemokines and respective receptors are involved in T cell attraction and inside-out signaling. However, Th1 cells are also able to bypass extracellular chemokine signals by absorbing chemokines stored intraendothelially in vesicles via dense lymphocyte-endothelial synapses (319). Moreover, EC also seem to be able to act in an APC like manner, presenting antigens specifically to memory T cells via their T cell receptor (TCR) and thereby activating them toward tissue migration (320). Whether these scenarios are also relevant for recruitment into atherosclerotic vessels is thus far not known.

Adhesion of the now activated T lymphocytes occurs presumably via the β2-integrin LFA-1, whereby VLA-4 and CD47 are also thought to play a role (79, 321). Similar to monocytes, crawling T cells interact with ICAM-1 via LFA-1 (322), polarize into a leading edge and tailing uropod and probe the endothelium by invasive protrusions (323) which is controlled by RhoA and continuous actin reorganization (324). The transmigration process of T cells in atherosclerotic arteries to date is mostly unknown, but according to T cell migration into other peripheral inflamed tissues likely similar to other leukocytes, favoring paracellular migration involving ICAM-1-mediated signaling and VE-cadherin dissociation from VE-PTP (325, 326). In contrast, TCR-activated effector memory CD4+ cells can also transmigrate in an alternative way involving CX3CL1 and LBRC adhesion proteins *in vitro* (327). Again, if this also happens in transmigration through atherosclerotic arteries, is not known.

### Platelets: Small but Effective Partners in Crime

Although far from giving a full picture, the role of platelets in atherosclerosis is becoming increasingly clear, revealing that their involvement extends well-beyond thrombus formation. In fact, thrombocytes also contribute to leukocyte recruitment in early atherogenesis, as will be elucidated in the following.

While shear stress can trigger endothelial activation, leading to upregulation of adhesion molecules and chemokines (see section Keep it Flowing), shear stress is also known to directly trigger platelet activation and aggregation in atherosclerotic vessels, thus promoting thrombotic arterial occlusion (328). However, shear stress or shear activated EC can stimulate platelets also in earlier phases of atherogenesis and promote platelet adhesion to the EC surface via enhanced adhesion molecule expression or decreased release of NO and prostacyclin (329), thus shifting the balance between inhibitory and activating pathways in platelets in favor of platelet activation. Of interest, impaired function of the ATP-binding cassette transporter G4 in bone marrow megakaryocyte progenitors giving rise to platelets has been shown to inhibit cholesterol efflux from these cells, thus promoting platelet production and accelerating atherosclerosis (330). Additionally, oxLDL was also shown to directly activate platelets via binding to CD36, thereby impairing cGMP mediated anti-inflammatory effects (331), while the traditional risk factors hyperlipidaemia, hyperglycaemia and hypertension were likewise associated with increased platelet reactivity (332).

Upon activation, platelets undergo shape change and increase surface expression of P-selectin and CD40L, while integrin αIIbβ3 adopts its active conformation (333). Such activated circulating platelets were shown to readily bind to EC and monocytes, deposit the chemokines CCL5 and CXCL4 (also known as PF4) on both EC and monocytes and subsequently promote leukocyte accumulation and atherosclerotic lesion formation in the arterial intima, notably already prior to the development of manifest atherosclerotic lesions (25, 334). Moreover, atherosclerotic plaques also suggest presence of macrophage-platelet aggregates, indicating that platelet-binding to monocytes persists beyond the recruitment process (335). In this interplay, platelets induce a pro-inflammatory phenotype in macrophages characterized by increased production of the cytokines IL-6 and IL-1β and impaired capability to phagocytose dying cells, which in turn results in increased necrotic core area in atherosclerotic plaques of *Ldlr*^−/−^ mice. In line, the amount of circulating monocyte-platelet aggregates was significantly increased in CAD patients (336, 337). Mechanistically, platelet binding to EC is a two-step process initiated by platelet rolling on the glycoprotein von Willebrand factor (vWF) released and bound by the endothelium, which involves platelet glycoprotein (GP)Ibα and P-selectin. Subsequently, firm adhesion of platelets is mediated via integrin αIIbβ3 which binds to endothelial αvβ3 and ICAM-1 (338), and involves PECAM-1 signaling (339). The interaction between activated platelets and leukocytes is conveyed by P-selectin interaction with leukocyte PSGL-1, which is supported by platelet glycoprotein Ibα (GPIbα), JAM-A, and JAM-C binding to leukocyte Mac-1 (340–342). In this context, the role of JAM-A is striking, as it seems to act as a brake on platelet activation: not only do knockout mice deficient for JAM-A display enlarged thrombi (343) and accelerated early-stage neointima formation (342), but also a hyperreactive phenotype significantly aggravating atherosclerotic lesion formation (344). However, in sharp contrast to these previous findings, a peptide antagonist intended to inhibit JAM-A function was shown to exert beneficial effects in atherosclerotic *Apoe*^−/−^ mice by inhibiting platelet adhesion to the endothelium (228). Therefore, further studies are warranted to clarify a possible correlation of these findings.

Strikingly, endothelium-adherent platelets were observed to form exceptionally long, flow-induced protrusions (FLIPR) from their membrane under high shear stress. Mediated by P-selectin/PSGL-1-interaction, such FLIPR can deliver platelet microvesicles to rolling monocytes and neutrophils which promotes their activation, as demonstrated by increased CD11b expression and L-selectin shedding (345). Another interesting observation is that, although platelets can adhere to EC at various shear rates *in vitro*, their ability to capture leukocytes may be limited to regions of disturbed flow (346). Moreover, the absence of platelets in mice markedly suppresses neutrophil crawling, whereas depletion of their neutrophil ligand PSGL-1 significantly alters neutrophil surface distribution of Mac-1 and CXCR2, thereby impairing directed intravascular motility and transmigration (269). On the other hand, platelets were also shown to recruit to atherosclerotic plaques by interacting with previously adhered monocytes and neutrophils in form of secondary capture (260).

Apart from favoring leukocyte recruitment by direct binding, several pro-inflammatory mediators released from activated platelets also promote leukocyte recruitment (332). One such platelet-derived chemokine of pivotal relevance to leukocyte recruitment is CCL5. It is deposited on activated EC in arteries mainly by platelets and significantly involved in monocyte and neutrophil adhesion to EC, as shown by *in vitro* and *in vivo* experiments (243, 347). Antagonizing CCL5, in turn, reduced neointima formation, leukocyte infiltration and atherosclerotic plaque formation in mice (348–350). CCL5 was also shown to form complexes with CXCL4 which synergistically enhances the capacity of CCL5 to recruit monocytes (351). Strikingly, this interaction could be selectively disrupted by the peptidic inhibitor CKEY2 and its mouse ortholog MKEY, thus decreasing monocyte recruitment and atherosclerotic plaque formation in mice (352). Another such liaison was found between CCL5 and the neutrophil-derived protein human neutrophil peptide 1 (HNP1), also facilitating monocyte recruitment to sites of inflammation. This could likewise be inhibited by application of the peptide antagonist RRYGTSKYQ (254).

A further important platelet derived chemokine is CXCL4 which is highly abundant in platelet α-granules and plays a critical role in coagulation. Importantly, CXCL4 could be localized in human atherosclerotic lesions and its presence on EC and macrophages positively correlated with clinical parameters for atherosclerosis (353). Moreover, CXCL4 was shown to directly bind oxLDL and to increase its uptake in vascular cells and macrophages (354), which is supported by histological findings in human atherosclerotic lesions (355). Also, upon stimulation with CXCL4, macrophages abolish expression of the atheroprotective scavenger receptor CD163 (356) and, as suggested by a recent study, give rise to a new macrophage phenotype called M4, characterized by the simultaneous expression of MMP7 and S100A8 (357). Additionally, CXCL4 appears to prompt differentiation of monocytes to macrophages (358). In line with these observations, depletion of CXCL4 in mice significantly reduced atherosclerotic lesion formation (359).

Another platelet derived factor is CXCL12. Interestingly, polymorphisms within the gene encoding for *CXCL12* in humans have been genome-wide significantly associated with CAD (360, 361). CXCL12 can signal both via CXCR4—relevant for neutrophil retention in the bone marrow—and CXCR7. While CXCR7 is barely detectable on blood leukocytes, it appears to be upregulated during monocyte-to-macrophage differentiation. This was accompanied by a switch in intracellular signaling in response to CXCL12 toward more pro-inflammatory pathways and subsequently enhanced phagocytotic activity of macrophages (362). While CXCL12 promotes monocyte chemotaxis in a CXCR4-dependent manner, monocyte adhesion to platelet-bound CXCL12 is rather mediated by CXCR7 (363). Furthermore, in a paracrine manner, CXCL12 was also shown to regulate platelet activation (364, 365). In line, *Cxcl12* overexpression increased, while endothelial-cell specific *Cxcl12* depletion reduced atherosclerotic lesion area in mice (366, 367) and platelet surface CXCL12 expression correlated with the risk of adverse cardiac events in symptomatic CAD patients undergoing percutaneous coronary intervention (PCI) (368). To the several other mediators released from platelets upon activation belong CXCL3, CXCL5, CXCL7, CXCL16, CCL3, and macrophage migration inhibitory factor (MIF). Noteworthy, platelets also release several angiogenesis-related proteins such as vascular endothelial growth factor (VEGF) or platelet derived growth factor (PDGF) with important influence on atherosclerosis. While angiogenic factors do not necessarily appear to influence leukocyte recruitment directly, neovascularization within atherosclerotic plaques is a hallmark of progressive atherosclerosis exponentially expanding the area over which leukocytes can penetrate, hence further promoting leukocyte infiltration and plaque destabilization (369).

### Lessons Learned: Clinical Implications and Therapeutic Options

Plasma levels of cholesterol, which circulates in the blood via LDL and strongly stimulates atherogenesis, can be successfully lowered by treatment with statins—an approach representing the mainstay of atherosclerosis therapy today. Further pharmacological strategies to prevent cardiovascular events include antihypertensive and antihyperglycemic agents, if applicable (370). However, according to a 2010 meta-analysis, average statin therapy in randomized, controlled trials (RCT) still leaves a mean residual risk of over 75% for major cardiovascular events in these patients (371). Intensified statin treatment and additional use of ezetimibe or PCSK9 inhibitors, which reduce levels of circulating LDL by a different mechanism, further strongly reduces this risk but, again, far from completely abolishing it (372, 373). Therefore, it becomes clear that fighting only the traditional risk factors is not sufficient to eliminate atherosclerosis.

**Figure 2:**
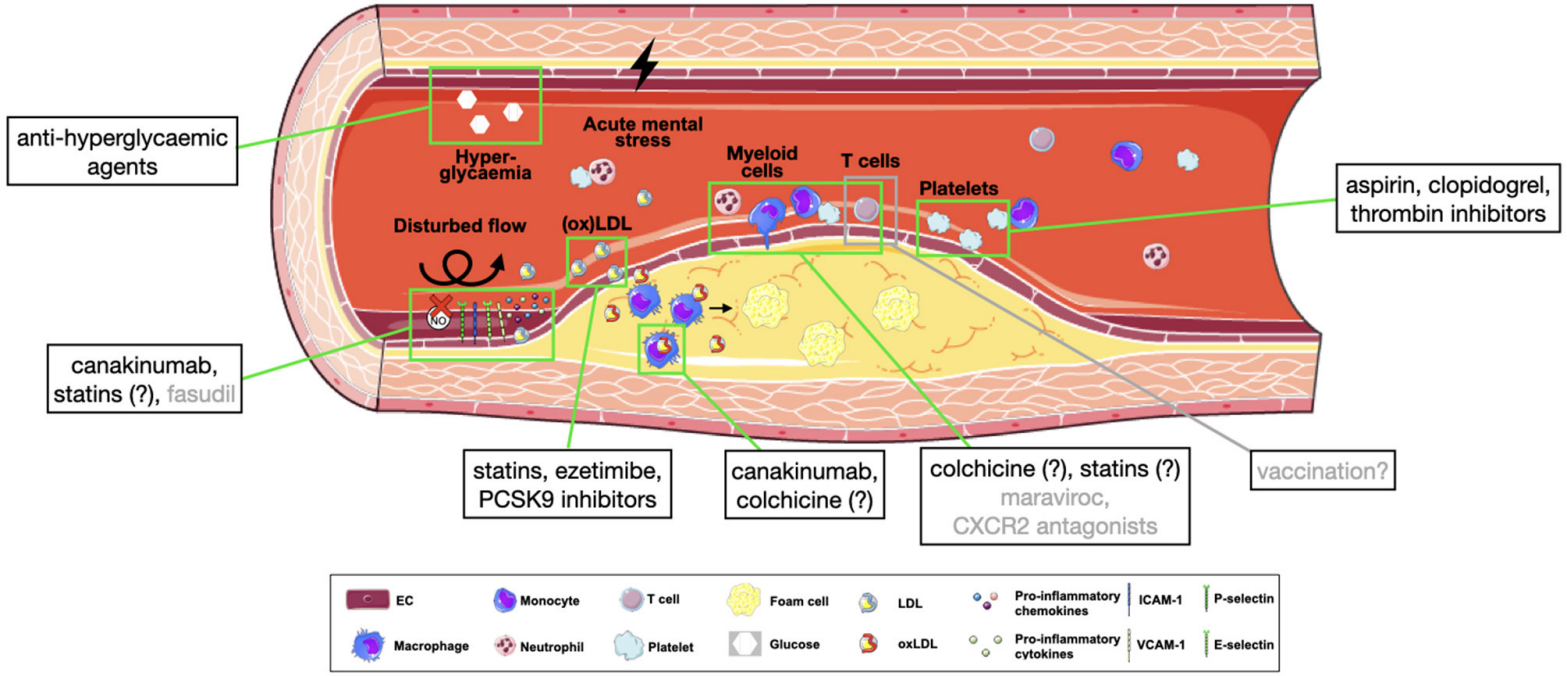
Pharmacological targets in atherosclerotic leukocyte recruitment. Simplified overview of approved (black) or potential (gray) targets in the process of leukocyte recruitment into atherosclerotic plaques. Substances with an incompletely clarified mechanism of action are marked with (?). CXCL2, C-X-C motif chemokine ligand 2; ICAM-1, intracellular adhesion molecule 1; ICAM-2, intracellular adhesion molecule 2; NO, nitric oxide; (ox)LDL, (oxidized) lipoprotein; PCSK9, proprotein convertase subtilisin/kexin type 9; VCAM-1, vascular adhesion molecule 1.

At this point, anti-inflammatory treatment strategies come to play (Figure 2). The IL-1β neutralizing antibody canakinumab was one of the first solely anti-inflammatory drugs shown to reduce the recurrence of cardiovascular events in high-risk CAD patients (374), and as suggested from mouse experiments, particularly does so by reducing leukocyte production in the bone marrow and deactivating EC toward less leukocyte recruitment (375). Moreover, another such highly anti-inflammatory drug, colchicine, was proven to reduce the risk of myocardial infarction, ischemic stroke, or cardiovascular death by over 25%, of note in addition to baseline treatment with lipid-lowering agents in 97% of enrolled patients (376). Colchicine's mechanism of action is not fully understood, but suggested to inhibit inflammasome activation, neutrophil recruitment and leukocyte-platelet interactions (377), thus directly affecting leukocyte migration into atherosclerotic plaques.

Of great interest, statins exert anti-inflammatory effects beyond their action on LDL (378), which may partly explain their superiority in cardiovascular risk reduction compared with other lipid-lowering agents. Mechanistically, in absence of hypercholesterolaemia, statins were shown to improve endothelial function, particularly by improving NO availability, stability of adherens junctions and reducing ROS formation (379–381), to inhibit neovascularization (382, 383), and, importantly, to selectively block LFA-1 and subsequent lymphocyte adhesion (384). Similarly, antithrombotic agents—a major pillar of secondary prevention—do not only reduce aggregation, but also platelet activation. Aspirin, for example, inhibits GPIIb/IIIa and P-selectin expression and release of chemokines (385, 386), clopidogrel was shown to improve systemic NO bioavailability and reduce soluble CD40L and CCL5 release (387) and thrombin inhibitors reduce formation of platelet-leukocyte aggregates and atherosclerotic plaques in mice (388, 389). Also some anti-hyperglycaemic agents have shown to exert beneficial effects on cardiovascular outcomes, both in diabetic and non-diabetic patients with heart failure (390, 391). Of note, the mechanism of action of some of these agents may affect NLRP3 inflammasome function in macrophages (392).

Up until now, strategies aimed at reducing vascular oxidative stress have not been shown beneficial in atherosclerosis patients (137). Interestingly, however, the Rho kinase inhibitor fasudil was associated with enhanced NO bioavailability, thus improving endothelial function in atherosclerotic patients (393). When regarding chemokine receptors, CXCR2 antagonists have been or are currently investigated in pilot or phase II studies in inflammatory diseases and COPD, while the CoronAry heart DiseAse (CICADA) study is specifically testing the cardiovascular effects of such agent (394). The CCR5 inhibitor maraviroc was shown to reduce aortic plaque size when treating atherosclerotic mice (395) but also to decrease atherosclerosis progression in HIV patients (396) and thus represents another interesting target for further studies.

A major fly in the ointment, however, is that large-scale inhibition of adhesion molecules or chemokines is often a double-edged sword, as several key players in leukocyte recruitment act differently in different tissues or have different, sometimes conflicting effects on atherosclerosis, as is the case with PECAM-1 (397). Another drawback is the importance of leukocyte recruitment for fighting infections. This limits therapeutical benefits of canakinumab, e.g., (374). Therefore, in contrast to targeting LDL, ubiquitous inhibition of adhesion molecules or chemokines is often not feasible. However, chrono-pharmacological treatment is one example for a more targeted approach: In mice, CCL2-dependent myeloid cell recruitment to atherosclerotic plaques peaks in the early morning and could be effectively targeted by time-adjusted treatment—importantly, without affecting cell adhesion in the cremasteric microcirculation at the same time (398). Another nascent concept is vaccination in atherosclerosis, aiming at inducing antigen-specific regulatory T cells to suppress deleterious effector T cell expansion and thus inhibit atherosclerosis. However, it is left open whether this strategy is feasible and beneficial (286).

Thus, it remains exciting to see what new therapeutics or therapeutic concepts will emerge in the future that prove helpful in curbing the inflammatory aspect of atherosclerosis and, in particular, leukocyte recruitment into the vessel walls.

## Conclusions

By following leukocytes step by step on their way into atherosclerotic plaques, it became clear that all leukocytes, although differing in their affinity for specific adhesion molecules or chemokines, use the same overall concept of tethering and rolling, adhesion, crawling and transmigration. Rolling is mainly mediated by PSGL-1 and during rolling, chemokine-chemokine receptor interactions activate the high affinity conformation of leukocyte integrins in a process called inside-out signaling, paving the way for firm adhesion via VLA-4 or LFA-1. Many chemokines have different affinities for different leukocytes, e.g., CCL2 rather favors classical monocytes and CXCL1 and 2 neutrophils, which may allow for targeted recruitment of a particular cell type. Other chemokines such as CCL5 are similarly important for monocytes, neutrophils and T cells, and are partially derived from platelets, which also directly contribute to leukocyte migration by binding to ECs and leukocytes. The concept of crawling and transmigration, however, appears to be very similar between leukocytes, involving cup formation and breaking of endothelial junctions. An important part of the recruitment process is EC priming, which enables expression or upregulation of adhesion factors such as P-selectin, ICAM-1 or VCAM-1 on ECs as binding partners for leukocyte ligands. ECs are mainly activated by two mechanisms: Disturbed flow and inflammatory mediators, most importantly oxLDL. Recent research shows important and divergent contributions of the different leukocyte subsets on plaque formation, with neutrophils being among the first cells to invade, paving the way for monocyte migration which, inside the plaque, differentiate to macrophages, ingest oxLDL, and promote further recruitment of leukocytes. T cells, on the other side, might contribute to atherosclerosis by targeting specific—mainly unidentified—antigens within the plaque.

Therapeutically targeting recruitment related processes is mainly drawn back by the multitude of important functions that most involved factors have in the immune system. Nevertheless, canakinumab and colchicine, two anti-inflammatory agents contributing to recruitment-related processes, were already proven beneficial in CAD patients. And surprisingly, statins, the mainstay of current atherosclerosis therapy, appear to not only lower LDL levels but also to inhibit leukocyte migration by affecting endothelial and leukocyte function. The same holds true for antithrombotic agents, which affect leukocyte recruitment in multiple ways. Many ongoing studies are investigating the effect of potential additional treatment strategies that mainly target the inflammatory nature of atherosclerosis. However, several open questions show that there is still a long way to go in basic research and on the road from bench to bedside, before we can control the excess inflammatory recruitment of leukocytes in atherosclerotic plaques in patients.

## Author Contributions

CM, JH, and HBS conceptualized the content, reviewed literature, wrote the manuscript, and generated figures. HS and TK discussed and edited the review. All authors contributed to the article and approved the submitted version.

## Funding

HBS has received funding from the European Research Council under the European Union's Horizon 2020 Research and Innovation Programme (STRATO, Grant Agreement No. 759272), the Else-Kröner-Fresenius-Stiftung (2020_EKSE.07), Else-Kröner-Forschungskolleg München, Technical University Munich, the Deutsche Herzstiftung (F/28/17), and the Deutsche Forschungsgemeinschaft (DFG) (SA 1668/5-1). TK was funded by the Corona Foundation as part of the Junior Research Group Translational Cardiovascular Genomics (S199/10070/2017) and the German Research Foundation (DFG) as part of the collaborative research center SFB 1123 (B02).

## Conflict of Interest

The authors declare that the research was conducted in the absence of any commercial or financial relationships that could be construed as a potential conflict of interest.

## Publisher's Note

All claims expressed in this article are solely those of the authors and do not necessarily represent those of their affiliated organizations, or those of the publisher, the editors and the reviewers. Any product that may be evaluated in this article, or claim that may be made by its manufacturer, is not guaranteed or endorsed by the publisher.

## Abbreviations

AGE: advanced glycosylation end product
ApoB-100: apolipoprotein B 100
*Apoe* ^−/−^: apolipoprotein E knockout
CAD: coronary artery disease
CCL: C-C motif chemokine ligand
CCR: C-C motif chemokine receptor
CD: cluster of differentiation
cGMP: cyclic guanosine monophosphate
CICADA: CoronAry heart DiseAse study
COPD: chronic obstructive pulmonary disease
CX3CL1: C-X3-C motif chemokine ligand 1
CX3CR1: C-X3-C motif chemokine receptor 1
CXCL: C-X-C motif chemokine ligand
CXCR: C-X-C motif chemokine receptor
EC: endothelial cells
eNOS: endothelial nitric oxide synthase
ESL-1: endothelial selectin ligand 1
F-actin: filamentous actin
FLIPR: flow-induced protrusions
FOXP3: forkhead box protein P3
GPCR: G-protein-coupled receptor
GPIbα: glycoprotein Ibα
GWAS: genome-wide association study
ICAM-1/-2/-3: intercellular adhesion molecule 1/2/3
IL: interleukin
JAM-A/-C: junctional adhesion molecule
Klf2: Krüppel-like factor 2
LBRC: lateral border recycling compartments
LDL: low density lipoprotein
*Ldlr* ^−/−^: low density lipoprotein receptor knockout
LFA-1: lymphocyte function-associated antigen 1
LOX-1: lectin-like oxidized LDL receptor 1
Ly6C: lymphocyte antigen 6 complex, locus C
Mac-1: macrophage receptor 1
MMP: matrix metalloproteinase
NET: neutrophil extracellular traps
NF-κB: nuclear factor κB
NLRP3: NOD-, LRR- and pyrin domain-containing protein 3
NO: nitric oxide
*NOS3*: nitric oxide synthase 3 (= eNOS) gene
Nrp1: neuropilin 1
oxLDL: oxidized low density lipoprotein
PAMP: pathogen-associated molecular patterns
PECAM-1: platelet/endothelial cell-adhesion molecule 1
PCSK9: proprotein convertase subtilisin/kexin type 9
PSGL-1: platelet selectin glycoprotein ligand 1
Rac1: Ras-related C3 botulinum toxin substrate 1
ROCK: Rho-associated protein kinase
ROS: reactive oxygen species
S100A8/9: S100 calcium-binding protein A8
sLox-1: soluble lectin-like oxidized LDL receptor 1
sGC: soluble guanylyl cyclase
SGEF: SH3-containing guanine nucleotide exchange factor
SNP: single nucleotide polymorphism
TAZ: transcriptional coactivator with PDZ-binding motif
TCR: T cell receptor
Th1/2/17: T helper cell subtype 1, 2, 17
TLR: Toll-like receptor
TNFα: tumor necrosis factor alpha
Treg: regulatory T cell
Trio: triple functional domain protein
VCAM-1: vascular cell adhesion molecule 1
VE-cadherin: vascular endothelial cadherin
VEGFR: vascular endothelial growth factor receptor
VE-PTP: vascular endothelial protein tyrosine phosphatase
VLA-4: very late antigen 4
YAP: yes-associated protein.
